# Myeloid Cell-Restricted Insulin Receptor Deficiency Protects Against Obesity-Induced Inflammation and Systemic Insulin Resistance

**DOI:** 10.1371/journal.pgen.1000938

**Published:** 2010-05-06

**Authors:** Jan Mauer, Bhagirath Chaurasia, Leona Plum, Thomas Quast, Brigitte Hampel, Matthias Blüher, Waldemar Kolanus, C. Ronald Kahn, Jens C. Brüning

**Affiliations:** 1 Department of Mouse Genetics and Metabolism, Institute for Genetics, University of Cologne, Cologne, Germany; 2 Center of Molecular Medicine Cologne (CMMC), Cologne, Germany; 3 Molecular Immune and Cell Biology Unit, Life and Medical Science Institute (LIMES), Bonn, Germany; 4 Department of Medicine, University of Leipzig, Leipzig, Germany; 5 Joslin Diabetes Center, Harvard Medical School, Boston, Massachusetts, United States of America; 6 Cologne Excellence Cluster on Cellular Stress Responses in Aging Associated Diseases (CECAD), University of Cologne, Cologne, Germany; 7 Second Department for Internal Medicine, University Hospital of Cologne, Cologne, Germany; 8 Max Planck Institute for the Biology of Ageing, Cologne, Germany; Stanford University School of Medicine, United States of America

## Abstract

A major component of obesity-related insulin resistance is the establishment of a chronic inflammatory state with invasion of white adipose tissue by mononuclear cells. This results in the release of pro-inflammatory cytokines, which in turn leads to insulin resistance in target tissues such as skeletal muscle and liver. To determine the role of insulin action in macrophages and monocytes in obesity-associated insulin resistance, we conditionally inactivated the insulin receptor (IR) gene in myeloid lineage cells in mice (IR^Δmyel^-mice). While these animals exhibit unaltered glucose metabolism on a normal diet, they are protected from the development of obesity-associated insulin resistance upon high fat feeding. Euglycemic, hyperinsulinemic clamp studies demonstrate that this results from decreased basal hepatic glucose production and from increased insulin-stimulated glucose disposal in skeletal muscle. Furthermore, IR^Δmyel^-mice exhibit decreased concentrations of circulating tumor necrosis factor (TNF) α and thus reduced c-Jun N-terminal kinase (JNK) activity in skeletal muscle upon high fat feeding, reflecting a dramatic reduction of the chronic and systemic low-grade inflammatory state associated with obesity. This is paralleled by a reduced accumulation of macrophages in white adipose tissue due to a pronounced impairment of matrix metalloproteinase (MMP) 9 expression and activity in these cells. These data indicate that insulin action in myeloid cells plays an unexpected, critical role in the regulation of macrophage invasion into white adipose tissue and in the development of obesity-associated insulin resistance.

## Author Summary

Obesity represents a major health burden with steadily increasing incidence. While it is associated with numerous co-morbidities, type 2 diabetes mellitus represents one of the major life-threatening, obesity-related conditions. Over the last years, it has become clear that during the course of obesity development not only does fat mass increase, but also fat composition changes qualitatively, leading to an influx of inflammatory cells, such as macrophages, into adipose tissue. Macrophages in turn secrete inflammatory mediators, which inhibit insulin action in skeletal muscle, liver, and even the central nervous system to ultimately cause insulin-resistant diabetes mellitus. However, the effect of insulin action and resistance in these inflammatory cell types themselves has not been addressed. To this end, we have generated and analyzed mice with inactivation of the insulin receptor specifically in myeloid cell-derived, inflammatory cells. Surprisingly, these animals are protected from the development of obesity-associated deterioration of glucose metabolism, thereby defining insulin action in inflammatory cells as a novel and promising target for therapeutic intervention against obesity-associated diabetes mellitus.

## Introduction

Obesity in humans and rodents is associated with increased expression of pro-inflammatory cytokines, such as tumor necrosis factor (TNF) α, in white adipose tissue (WAT) [1]–[4]. This results from increased cytokine expression in WAT and more importantly from infiltration of WAT by macrophages [5]–[7]. Elevated concentrations of these cytokines activate the c-Jun N-terminal kinase (JNK)-, nuclear factor (NF) kB- and Jak/Stat/Socs-signaling pathways in metabolic target tissues of insulin action such as skeletal muscle and liver, thereby inhibiting insulin signal transduction [8]–[10]. Inactivation of the inhibitor of NFkB kinase beta (IKK2), the main activator of TNF-α-stimulated NFkB activation in myeloid cells, protects mice from the development of obesity-associated insulin resistance [11]. These findings suggest that macrophages play a key role in the development of obesity-associated insulin resistance and type 2 diabetes. More recently, a critical role in the development of obesity-associated inflammation has also been demonstrated for mast cells and lymphocytes [12], [13].

Early studies indicated that macrophages and monocytes express insulin receptors [14], however, the physiological function of these receptors has been a matter of debate. Macrophages and monocytes have been shown to respond to insulin with increased phagocytosis and glucose metabolism [15] and with increased TNF-α production and inhibition of apoptosis [16], [17]. Additionally, it has been reported that bone marrow-specific deletion of cbl-associated protein (CAP), a downstream molecule of the insulin signaling cascade, protects mice against obesity-induced insulin resistance [18]. To directly address the role of insulin action and resistance in myeloid cells, we generated mice with cell type-specific deletion of the insulin receptor in this lineage (IR^Δmyel^-mice). We have previously reported that these animals, upon exposure to a high cholesterol diet, exhibit protection from the development of atherosclerosis in the presence of reduced inflammation on an apolipoprotein E (ApoE)-deficient background [19]. However, others reported more complex lesions in the absence of myeloid cell insulin action through activation of the endoplasmatic reticulum (ER) stress pathway on a low-density lipoprotein receptor (LDLR)-deficient background [20]. Nonetheless, these studies did not address the role of insulin action and insulin resistance in myeloid lineage cells under conditions of obesity and obesity-induced inflammation and insulin resistance. To analyze the impact of myeloid cell-restricted insulin resistance on the development of systemic insulin resistance associated with obesity, we characterized glucose metabolism in control- and IR^Δmyel^-mice receiving either a normal chow diet or a high fat diet.

## Results

### Myeloid cell-restricted insulin resistance does not affect obesity development upon high fat feeding

**Figure 1:**
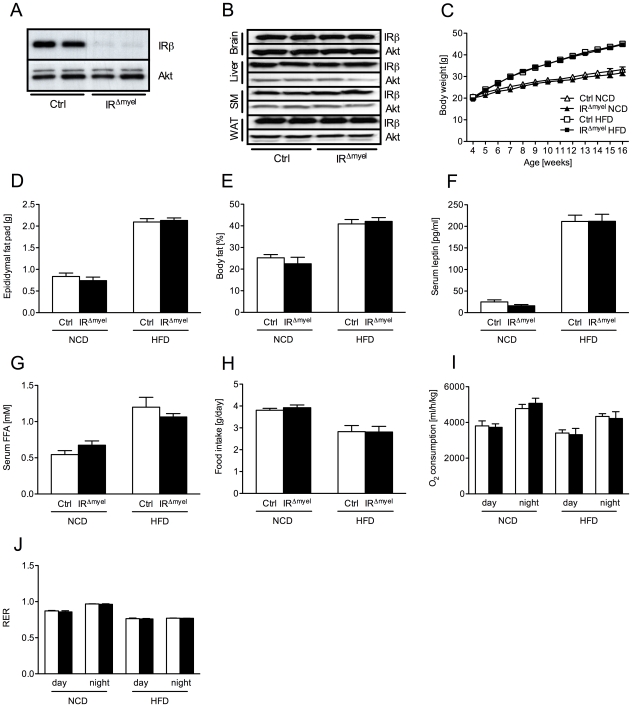
IR^Δmyel^-mice exhibit unaltered response to normal chow and high fat diet. (A) Western blot analysis of insulin receptor (IR) β and Akt (loading control) expression in thioglycollate-elicited macrophages of control- and IR^Δmyel^-mice. (B) Western blot analysis of IR-β and Akt (loading control) in brain, liver, skeletal muscle (SM) and white adipose tissue (WAT) of control- and IR^Δmyel^-mice. (C) Weight curves of male control- and IR^Δmyel^-mice fed NCD or HFD. (n = 12 mice per genotype on NCD; n = 32 mice per genotype on HFD.) (D) Epididymal fat pad mass of male control- and IR^Δmyel^-mice fed either NCD or HFD. (n = 15 mice per genotype and diet.) (E) Body fat content of male control- and IR^Δmyel^-mice fed either NCD or HFD. (n = 4–10 mice per genotype and diet.) (F) Serum leptin concentrations of male control- and IR^Δmyel^-mice fed either NCD or HFD. (n = 6–16 mice per genotype and diet.) (G) Serum free fatty acid (FFA) concentrations of male control- and IR^Δmyel^-mice fed either NCD or HFD. (n = 10–12 mice per genotype and diet.) (H) Daily food intake of male control- and IR^Δmyel^-mice fed either NCD or HFD. (n = 4–10 mice per genotype and diet.) (I) Oxygen (O_2_) consumption of male control- and IR^Δmyel^-mice fed either NCD or HFD. (n = 4–10 mice per genotype and diet.) (J) Respiratory exchange ratio (RER) of male control- and IR^Δmyel^-mice fed either NCD or HFD. (n = 4–10 mice per genotype and diet.) (Results are means ± SEM; white bars represent controls and black bars represent IR^Δmyel^-mice).

As previously shown, crossing IR^flox/flox^-mice with mice expressing the Cre-recombinase under control of the lysozymeM promoter resulted in efficient, myeloid cell-restricted ablation of the insulin receptor [19] (Figure 1A and 1B). Under normal chow diet (NCD), control- and IR^Δmyel^-mice exhibited indistinguishable weight curves, white adipose tissue mass, body fat content, serum leptin concentrations and serum free fatty acids (FFA) (Figure 1C–1G). When exposed to high fat diet (HFD), control-mice significantly gained weight over animals exposed to NCD, and the degree of weight gain was similar between control- and IR^Δmyel^-mice (Figure 1C). Moreover, white adipose tissue mass, body fat content, circulating leptin concentrations as an indirect measure of fat mass and serum FFA were significantly elevated in mice exposed to HFD, but indistinguishable between control- and IR^Δmyel^-mice (Figure 1D–1G). Additionally, food intake, oxygen (O_2_) consumption and respiratory exchange ratio (RER) were modulated by exposure to HFD but did not show any difference between both genotypes (Figure 1H–1J). Taken together, these results indicate that insulin receptors on myeloid cells are not required for energy homeostasis under NCD and HFD feeding and that myeloid cell-restricted insulin resistance does not affect the development of obesity upon high fat feeding.

### IR^Δmyel^-mice exhibit improved glucose metabolism upon high fat feeding

**Figure 2:**
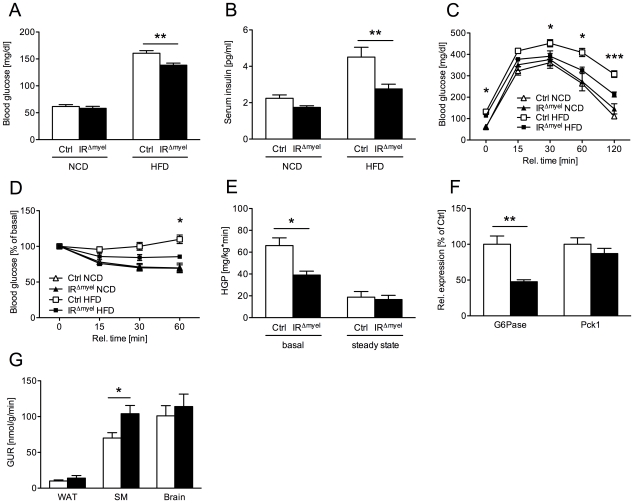
IR^Δmyel^-mice are protected against obesity-induced insulin resistance. (A) Fasted blood glucose concentrations of male control- and IR^Δmyel^-mice fed either NCD or HFD. (n = 6–7 mice per genotype on NCD; n = 33–36 mice per genotype on HFD.) (B) Fasted serum insulin concentrations of male control- and IR^Δmyel^-mice fed either NCD or HFD. (n = 6–7 mice per genotype on NCD; n = 10 mice per genotype on HFD.) (C) Glucose Tolerance Tests were performed with male control- and IR^Δmyel^-mice fed NCD or HFD. (n = 6–13 mice per genotype and diet.) (D) Insulin Tolerance Tests were performed with male control- and IR^Δmyel^-mice fed NCD or HFD. (n = 4–11 mice per genotype and diet.) (E) Hepatic glucose production (HGP) of male, HFD-fed control- and IR^Δmyel^-mice before (basal) and during (steady state) euglycemic, hyperinsulinemic clamp analysis. (n = 12 mice per genotype.) (F) Relative expression of G6Pase and Pck1 mRNA in livers of fasted control- and IR^Δmyel^-mice fed HFD (n = 6 mice per genotype.) (G) Tissue-specific glucose uptake rate (GUR) of male, HFD-fed control- and IR^Δmyel^-mice under steady state conditions. (WAT = white adipose tissue; SM = skeletal muscle; n = 10 mice per genotype.) (Results are means ± SEM; white bars represent controls and black bars represent IR^Δmyel^-mice; *p≤0.05; **p≤0.01; ***p≤0.001.)

To address the role of myeloid cell insulin action on whole body glucose metabolism, we next determined blood glucose and serum insulin concentrations in control- and IR^Δmyel^-mice. Both parameters were indistinguishable between genotypes under NCD (Figure 2A and 2B). As expected, on HFD, control-mice developed significantly increased blood glucose and serum insulin concentrations suggestive of insulin resistance (Figure 2A and 2B). Glucose and insulin levels did also rise in obese IR^Δmyel^-mice, but strikingly, this increase was significantly blunted in these mice lacking insulin receptors in myeloid cells (Figure 2A and 2B).

Consistent with this, glucose tolerance was similar in control- and IR^Δmyel^-mice under NCD and became impaired in control-mice administered a HFD (Figure 2C). In contrast, IR^Δmyel^-mice receiving the HFD demonstrated only a minimal impairment in glucose tolerance compared to control- or IR^Δmyel^-mice on NCD (Figure 2C). Similarly, obese IR^Δmyel^-mice showed significantly higher insulin sensitivity as measured by insulin tolerance test when compared to HFD-fed control-mice, whereas insulin sensitivity was comparable between both groups under NCD (Figure 2D). Taken together, these data reveal that myeloid cell-restricted insulin receptor deficiency leads to striking protection from obesity-induced insulin resistance.

### IR^Δmyel^-mice exhibit reduced hepatic glucose production and improved insulin action in skeletal muscle upon high fat feeding

To further define in which tissues myeloid cell-autonomous insulin resistance affects systemic glucose metabolism on HFD, we performed euglycemic, hyperinsulinemic clamps in control- and IR^Δmyel^-mice after 12 weeks of exposure to HFD. This analysis revealed a significant decrease in basal hepatic glucose production in IR^Δmyel^- compared to control-mice, while insulin-suppressed HPG (steady state) was similar in both groups (Figure 2E). Accordingly, obese IR^Δmyel^-mice exhibited a 50% reduction in the hepatic expression of a key enzyme of gluconeogenesis, glucose-6-phosphatase (G6Pase), while expression of phosphoenolpyruvate carboxykinase (Pck1) remained unchanged (Figure 2F).

In addition, insulin-stimulated glucose disposal in skeletal muscle was significantly increased in IR^Δmyel^- compared to control-mice, whereas insulin-stimulated glucose uptake in brain and adipose tissue remained unaltered under clamp conditions (Figure 2G).

In summary, these experiments indicate that the major improvement in glucose metabolism of obese IR^Δmyel^-mice results from both increased insulin sensitivity in skeletal muscle and reduced basal hepatic glucose production.

### Decreased systemic, obesity-associated inflammatory response in IR^Δmyel^-mice

**Figure 3:**
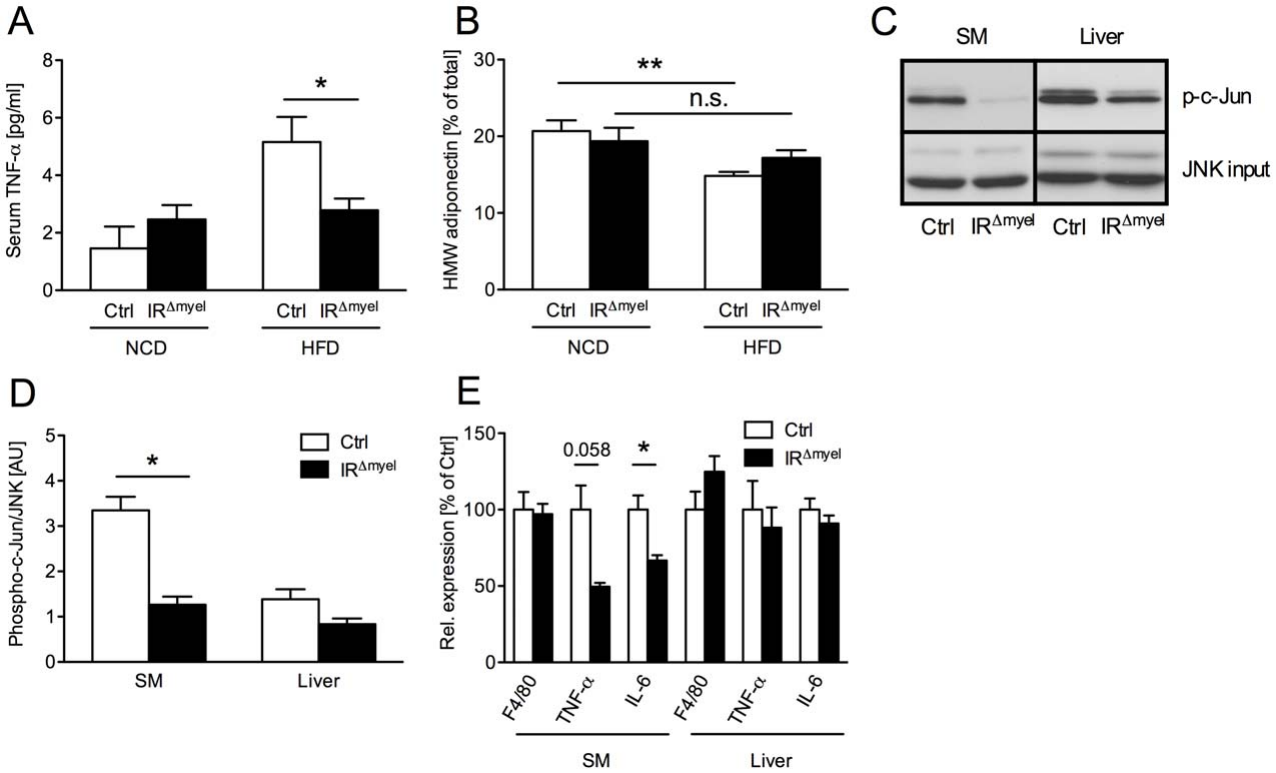
The obesity-associated systemic pro-inflammatory state is reduced in IR^Δmyel^-mice. (A) Serum TNF-α concentration in male control- and IR^Δmyel^-mice fed either NCD or HFD. (n = 12 mice per genotype on NCD; n = 21 mice per genotype on HFD.) (B) Percentage of serum high molecular weight (HMW) from total adiponectin in male control- and IR^Δmyel^-mice fed either NCD or HFD. (n = 10–12 mice per genotype and diet.) (C) *In vitro* phosphorylation of c-Jun (p-c-Jun) in skeletal muscle (SM) and liver lysates from male, HFD-fed control- and IR^Δmyel^-mice. Total JNK input was used as loading control. (representative western blot shown). (D) Densitometrical analysis of phospho-c-Jun *vs* total JNK. (AU = arbitrary units; SM = skeletal muscle; n = 6 mice per genotype.) (E) Relative expression of F4/80, TNF-α and IL-6 mRNA in skeletal muscle (SM) and liver of male, HFD-fed control- and IR^Δmyel^-mice. (n = 6 mice per genotype.) (Results are means ± SEM; white bars represent controls and black bars represent IR^Δmyel^-mice; *p≤0.05; **p≤0.01; n.s. = not significant.)

As insulin resistance in response to obesity and high fat feeding has been demonstrated to arise from increased concentrations of local and circulating pro-inflammatory cytokines [21] and from a reduction of circulating adiponectin concentrations [22], [23], we determined these parameters in control- and IR^Δmyel^-mice. Exposure of control-mice to HFD induced a marked increase of serum TNF-α concentrations compared to animals fed NCD. Strikingly, this obesity-induced increase in TNF-α was completely blunted in IR^Δmyel^-mice (Figure 3A). Moreover, while high fat feeding significantly reduced the portion of high molecular weight (HMW) adiponectin of total serum adiponectin in control animals, this diet-induced reduction was not observed in IR^Δmyel^-mice (Figure 3B).

Since increased concentrations of TNF-α have been demonstrated to activate inflammatory signaling cascades critical in the development of insulin resistance in classical insulin target tissues, we next directly investigated the activation of c-Jun N-terminal kinase (JNK) signaling in liver and skeletal muscle of obese control and IR^Δmyel^-mice. This analysis revealed, that basal JNK activity, as assessed by phosphorylation of c-Jun, was significantly reduced in skeletal muscle and exhibited a trend towards reduction in liver of obese IR^Δmyel^-mice compared to controls (Figure 3C and 3D).

Furthermore, expression of pro-inflammatory cytokines TNF-α and IL-6 was reduced in skeletal muscle, but not liver of obese IR^Δmyel^-mice (Figure 3E). However, the number of macrophages, which represent a major source for these cytokines, was unaltered in either tissue as demonstrated by similar expression of the macrophage-specific mRNA F4/80 in both groups of mice (Figure 3E).

Taken together, these experiments demonstrate that myeloid cell-restricted insulin resistance protects from obesity-associated systemic changes in the circulating concentrations of cytokines and adipokines as well as the local activation of JNK in skeletal muscle.

### Decreased macrophage recruitment and obesity-associated inflammation in white adipose tissue of IR^Δmyel^-mice

**Figure 4:**
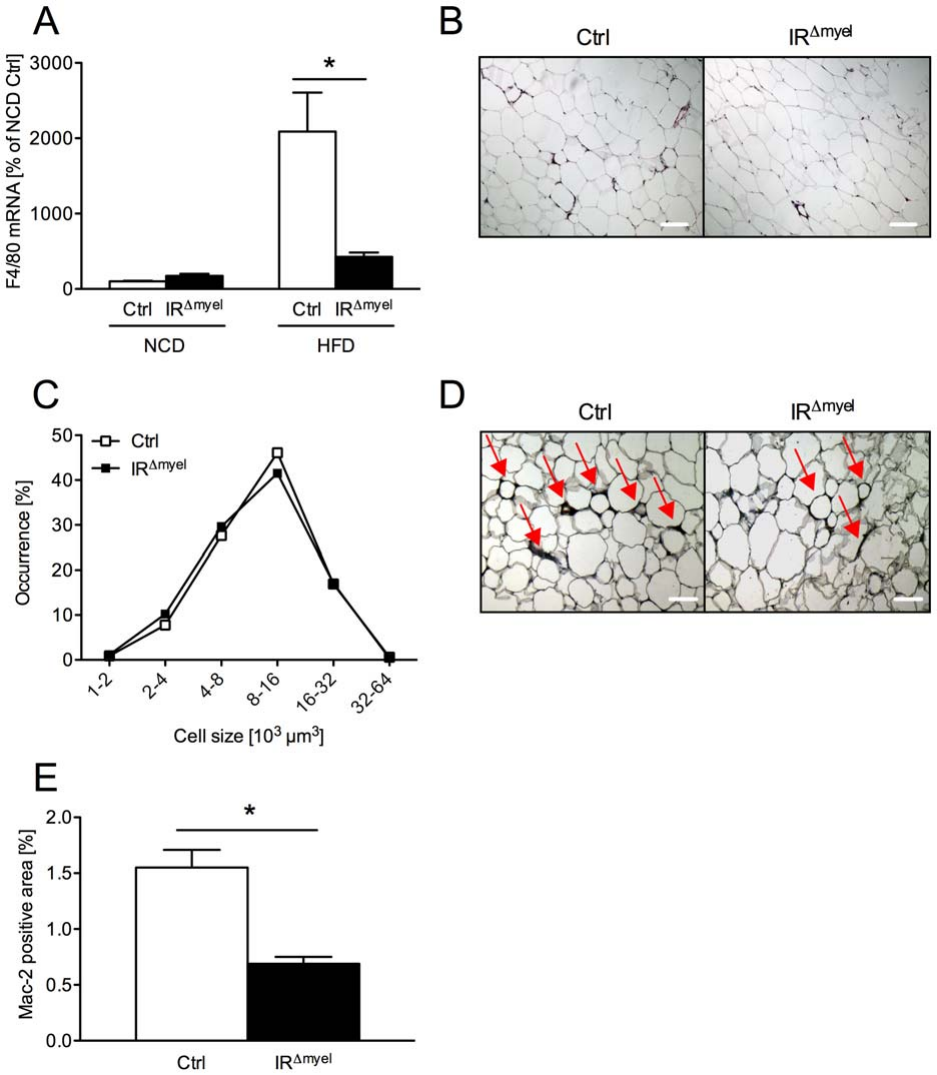
The obesity-associated macrophage infiltration into white adipose tissue is blunted in IR^Δmyel^-mice. (A) Relative expression of F4/80 mRNA in WAT of male control- and IR^Δmyel^-mice fed either NCD or HFD. (n = 8 mice per genotype and diet.) (B) Hematoxylin and eosin staining of white adipose tissue (WAT) sections from male control- and IR^Δmyel^-mice fed HFD. (C) Adipocyte size distribution in WAT of male control- and IR^Δmyel^-mice fed HFD. (n = 9 mice per genotype.) (D) Mac-2 staining of WAT-sections from male control- and IR^Δmyel^-mice fed HFD; Red arrows indicate Mac-2 positive area surrounding the adipocytes. (E) Percentage of Mac-2 positive area per section in male control- and IR^Δmyel^-mice fed HFD. (n = 9 mice per genotype.) (Results are means ± SEM; white bars represent controls and black bars represent IR^Δmyel^-mice; *p≤0.05; scale bars = 200 µm.)

To address whether the observed reduction in systemic, obesity- associated inflammatory response correlates with alterations of the local, obesity-associated infiltration of adipose tissue by macrophages, we next analyzed the expression of F4/80, a specific marker for this cell-type, in WAT of control- and IR^Δmyel^-mice by quantitative realtime PCR analysis. Compared to NCD, high fat feeding significantly enhanced expression of F4/80 mRNA in adipose tissue of control animals. Strikingly, the diet-induced increase of this marker was almost completely abolished in WAT of IR^Δmyel^-mice (Figure 4A). This decrease appeared to represent reduced macrophage recruitment, since bone marrow-derived macrophages (BMDM) of IR^Δmyel^-mice showed unaltered expression of F4/80 mRNA under basal conditions and after treatment with the saturated fatty acid palmitate compared to control cells (Figure S1A). Importantly, among classical insulin target tissues, only adipose tissue showed drastically increased diet-induced expression of F4/80 mRNA, while expression in obese liver and skeletal muscle was not significantly modulated in wildtype animals compared to NCD (Figure S1B).

Furthermore, we performed histological analyses of WAT obtained from obese control- and IR^Δmyel^-mice. No difference was observable in adipocyte morphology or adipocyte size distribution between control- and IR^Δmyel^-mice on high fat diet (Figure 4B and 4C), consistent with unaltered obesity development in these animals. However, in line with the reduced mRNA expression of macrophage marker genes, immunohistochemical analysis of infiltrating, activated macrophages demonstrated a striking reduction of Mac-2-positive cells in WAT of diet-induced obese IR^Δmyel^-mice compared to control-mice (Figure 4D and 4E). Thus, the reduced F4/80 mRNA and Mac-2 antigen expression in WAT in the presence of unaltered macrophage-autonomous marker gene expression clearly provides independent experimental evidence for reduced macrophage recruitment to WAT of IR^Δmyel^-mice upon high fat feeding.

**Figure 5:**
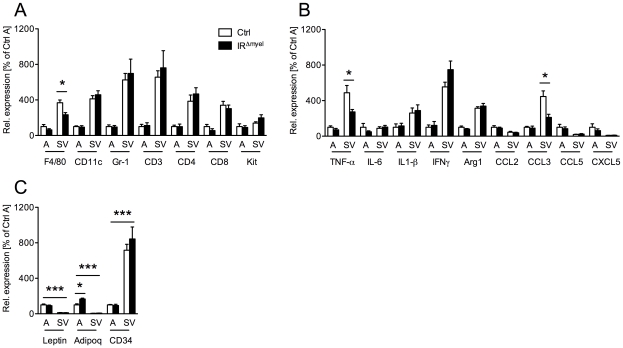
Obese IR^Δmyel^-mice exhibit reduced macrophage marker and pro-inflammatory gene expression in stromal vascular cells of the adipose tissue. (A) Relative expression of immune cell markers F4/80, CD11c, Gr-1, CD3, CD4, CD8 and Kit mRNA in adipocytes (A) and stromal vascular (SV) fraction of male control- and IR^Δmyel^-mice fed HFD. (n = 5 mice per genotype.) (B) Relative expression of cytokines and chemokines TNF-α, IL-6, IL-1β, IFNγ, Arg1, CCL2/MCP1, CCL3/MIP1α, CCL5/Rantes and CXCL5 mRNA in adipocytes (A) and stromal vascular (SV) fraction of male control- and IR^Δmyel^-mice fed HFD. (n = 5 mice per genotype.) (C) Relative mRNA expression of adipocyte-specific genes leptin and adiponectin (adipoq) and stromal vascular cell-specific gene CD34 in adipocytes (A) and stromal vascular (SV) fraction of male control- and IR^Δmyel^-mice fed HFD. (n = 5 mice per genotype.) (Results are means ± SEM; white bars represent controls and black bars represent IR^Δmyel^-mice; *p≤0.05; ***p≤0.001.)

Since not only macrophages, but also a variety of other immune cells are highly abundant in the obese adipose tissue and contribute to the development of obesity-induced insulin resistance [12], [13], [24], we assessed mRNA expression of different immune cell markers in the stromal vascular (SV) fraction of WAT from obese control- and IR^Δmyel^-mice. In control mice, markers for macrophages (F4/80), dendritic cells (CD11c), granulocytes (Gr-1), T-lymphocytes (CD3, CD4, CD8) and mast cells (Kit) were highly enriched in the SV fraction compared to adipocytes (Figure 5A). However, in line with the data on whole WAT, we observed a specific reduction of the macrophage marker F4/80, but not of markers for granulocytes, mast cells, dendritic cells or T-lymphocytes, in the SV fraction of IR^Δmyel^-mice (Figure 5A).

Consistent with the specific reduction of macrophage infiltration, further analysis revealed a decrease in mRNA expression of the cytokine TNF-α and the chemokine CCL3/MIP-1α in SV fraction of IR^Δmyel^-mice (Figure 5B), indicating reduced inflammation in this compartment. Notably, although TNF-α, interleukin (IL) 1β, interferon (IFN) γ and arginase (Arg) 1 showed higher expression in SV fraction than in adipocytes, IL-6, CCL2/MCP-1, CCL5/Rantes and CXCL5 were equally if not higher expressed by adipocytes compared to SV fraction (Figure 5B).

To verify efficient separation of adipocytes from SV fraction, we analyzed expression of leptin, adiponectin and CD34 in both compartments. As expected, leptin and adiponectin were exclusively expressed in adipocytes, while CD34 expression was highly restricted to the SV fraction (Figure 5C).

Importantly, adiponectin expression was significantly increased in adipocytes from IR^Δmyel^-mice compared to controls, pointing towards increased insulin sensitivity in these animals (Figure 5C).

Taken together, our data indicate that disruption of the insulin receptor in myeloid cells specifically interferes with the obesity-associated recruitment of macrophages to adipose tissue and ultimately leads to reduced local expression of cytokines and chemokines in WAT.

### Insulin receptor–deficient macrophages exhibit increased susceptibility to lipid-induced apoptosis and reduced expression of matrix metalloproteinase 9

The observed reduction of adipose tissue macrophage content in obese IR^Δmyel^-mice, among other factors, might arise from (i) enhanced susceptibility to apoptosis or (ii) reduced invasive capacity of these cells.

To directly address the hypothesis that IR signaling in macrophages might control these processes, we first analyzed the regulation of apoptosis in response to fatty acids to mimic the metabolic environment present upon high fat feeding. To this end, macrophages were isolated from the bone marrow of control- and IR^Δmyel^-mice, stimulated with palmitate in the absence or presence of insulin and TUNEL assays were performed. Palmitate stimulation profoundly induced apoptosis in control macrophages and insulin significantly reduced the number of TUNEL-positive cells in control cells both in the absence and presence of lipid stimulation (Figure S2A, S2B). However, insulin failed to reduce apoptosis in the IR-deficient macrophages in either the basal or palmitate-stimulated state (Figure S2A, S2B). Furthermore, quantitative realtime PCR analysis suggested that the protective effect of insulin is mediated through stimulation of Bcl-2 mRNA rather than suppression of Bax mRNA expression (Figure S2C, S2D). Nonetheless, this *in vitro* observation did neither translate into increased numbers of apoptotic macrophages in adipose tissue nor into reduced numbers of circulating monocytes in obese IR^Δmyel^-mice (data not shown).

**Figure 6:**
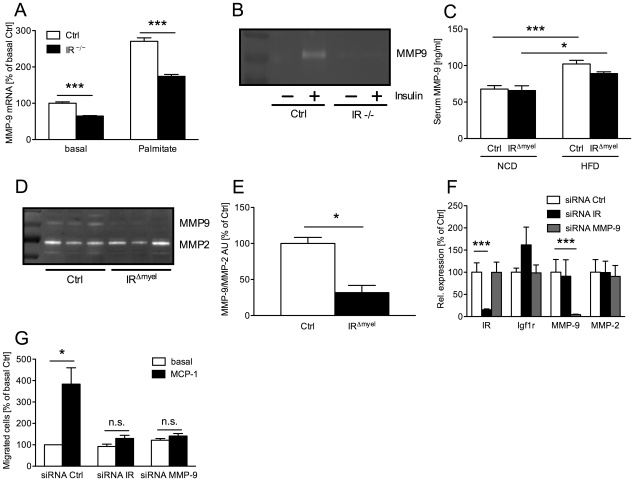
Myeloid cell-restricted insulin receptor deficiency leads to reduced matrix metalloproteinase (MMP) 9 expression in macrophages and white adipose tissue of IR^Δmyel^-mice. (A) Relative expression of MMP-9 mRNA in peritoneal macrophages of control- and IR^Δmyel^-mice. Cells were left untreated (basal) or stimulated with palmitate (500 µM) for 4 h. (n = 3 independent experiments.) (B) Conditioned medium from 24 h untreated (basal) and insulin-stimulated (50 ng/ml) bone marrow-derived macrophages (BMDM) of control- and IR^Δmyel^-mice (IR −/−) was analyzed for MMP-9 gelatinolytic activtity. (representative zymogram of 3 independent experiments shown.) (C) Serum MMP-9 concentration in male control- and IR^Δmyel^-mice fed either NCD or HFD. (n = 10–12 mice per genotype and diet.) (D) White adipose tissue lysates from obese control- and IR^Δmyel^-mice were analyzed for gelatinolytic activity. (E) Densitometrical analysis of MMP-9 *vs* MMP-2 in zymograms of WAT from obese control- and IR^Δmyel^-mice. (AU = arbitrary units; n = 3.) (F) Relative expression of insulin receptor (IR), insulin-like growth factor 1 receptor (Igf1r), matrix metalloproteinase (MMP) 9 and MMP-2 mRNA in silenced BMDM. (white bars = siRNA Ctrl, black bars = siRNA IR, grey bars = siRNA MMP-9; n = 3.) (G) Chemotaxis of silenced BMDM through a gelatin matrix was analyzed using a transwell migration assay. (white bars = basal migration, black bars = migration against 100 ng/ml MCP-1; n = 3 independent experiments.) (Results are means ± SEM; white bars represent controls and black bars represent IR-deficient macrophages/IR^Δmyel^-mice unless stated otherwise; *p≤0.05; ***p≤0.001; n.s. = not significant.)

Besides control of macrophage survival, an important prerequisite for macrophage invasion into tissues is their ability to express and secrete matrix metalloproteinases (MMPs), which then help to degrade extracellular matrix (ECM) proteins to allow trans-ECM migration. Since it has recently been established that MMP-9 (gelatinase B) plays a critical role in inflammatory macrophage migration [25], we assessed MMP-9 expression and activation in macrophages of control- and IR^Δmyel^-mice. Peritoneally elicited macrophages were either left untreated (basal) or were stimulated with palmitate and expression of MMP-9 mRNA was determined. Intriguingly, IR-deficient cells exhibited a dramatic reduction of MMP-9 mRNA expression both in the basal state as well as upon stimulation with palmitate (Figure 6A). Importantly, IR disruption in macrophages not only affected MMP-9 expression, but also translated into reduced MMP-9 activity. Thus, zymographical analysis of conditioned media revealed higher MMP-9 activity in those obtained from control macrophages compared to those from IR-deficient cells (Figure 6B).

To verify the *in vivo* relevance of this cell-autonomous impairment in MMP-9 expression and activation, we first determined serum MMP-9 concentrations in lean and obese control and IR^Δmyel^-mice. While HFD induced a highly significant increase of circulating MMP-9 in control animals, this increase was less profound in IR^Δmyel^-mice (Figure 6C). Furthermore, we assessed gelatinolytic activity in WAT lysates of obese control- and IR^Δmyel^-mice. Strikingly, while MMP-2 activation was unaltered, WAT of IR^Δmyel^-mice exhibited drastically reduced MMP-9 activation compared to controls, possibly reflecting the reduced accumulation of macrophages in this compartment (Figure 6D and 6E).

To further functionally analyze the effect of IR-deficiency on macrophage migration, we performed transwell migration assays with wildtype BMDM transfected with siRNAs directed against either IR or MMP-9. Compared to a scrambled control siRNA, both oligonucleotides mediated efficient and specific knockdown of their respective target mRNAs without reducing expression of closely related insulin-like growth factor (IGF) 1 receptor and MMP-2 mRNA (Figure 6F). BMDM transfected with the control siRNA showed an approximately 4-fold increase of migrated cells through gelatin-coated membranes in response to MCP-1 compared to the basal level (Figure 6G). However, knockdown of MMP-9 significantly blunted this response and MCP-1 failed to enhance basal migration significantly (Figure 6G). Strikingly, siRNA-mediated ablation of IR reduced macrophage migration capacity to a similar degree as that of MMP-9 (Figure 6G).

Taken together, our experiments reveal that insulin action in macrophages promotes tissue invasion capacity of these cells *in vitro* and *in vivo*, thereby critically controlling high fat diet-associated macrophage invasion and activation in WAT upon induction of obesity.

## Discussion

Insulin resistance in metabolically relevant insulin target tissues, such as skeletal muscle, liver, adipose tissue and more recently the brain, represents a well-studied key characteristic during the development of type 2 diabetes mellitus [26]–[30]. Insulin resistance can arise via different mechanisms e.g. mutations in genes encoding insulin signaling components or their reduced expression [31], [32]. However, it has been demonstrated that insulin resistance associated with obesity largely stems from posttranslational modifications of insulin signaling proteins, such as inhibitory serine phosphorylation of the insulin receptor or its downstream signaling mediators [33]. Here, activation of pro-inflammatory signaling cascades, particularly JNK and IKK, have been shown to inhibit insulin action, although to different, tissue-specific extent [8], [34]–[36]. The establishment of a chronic pro-inflammatory state during the course of obesity stems from expression of pro-inflammatory cytokines in adipose tissue, particularly through the recruitment of cells of the innate immune response system to WAT [5], [7]. The critical importance of innate immune response activation during the development of obesity-associated insulin resistance has been highlighted by the phenotype of mice with targeted disruption of the NFκB pathway in myeloid lineage cells, as well as mice deficient for the chemokine receptor CCR2, which both exhibit reduced WAT inflammation and are therefore protected from obesity-induced insulin resistance [11], [37].

While these findings have provided compelling evidence for the immune response pathway to cause insulin resistance in liver, skeletal muscle and adipose tissue, the primary effect of insulin action and insulin resistance in cells of the innate immune system has been poorly investigated and remains controversial. Thus, while there is considerable evidence for a role of inflammation in producing insulin resistance in individuals with type 2 diabetes [38]–[40], it has also been shown that insulin treatment of obese humans can reverse the pro-inflammatory state in macrophages [41], [42], raising a question of which is cause and which is effect. Also, it is not clear if this anti-inflammatory effect is a direct effect of insulin on cells of the immune system or if the reversal of inflammation occurs secondary to normalization of hyperglycemia and other metabolic abnormalities [43]. To further complicate the matter, insulin has been shown to directly increase TNF-α expression in human monocytes, pointing towards a possible direct pro-inflammatory role for insulin in macrophages [16]. Indeed, the latter is consistent with our previous observation that myeloid cell-restricted insulin resistance protects apolipoproteinE-deficient mice from the development of atherosclerosis due to impaired inflammatory response [19]. Alternatively, differential pro- and anti-inflammatory effects of insulin may represent different stages of a process representing acute *versus* chronic stimulation [44].

The findings of the present study directly demonstrate an unexpected and pivotal role for insulin signal transduction in the control of innate immune cell behavior in the obese state, such that chronic impairment of insulin action in myeloid lineage cells protects from obesity-associated inflammation. This is further supported by the observation that mice with bone marrow-restricted disruption of cbl-associated protein (CAP), a downstream component of insulin action in control of glucose transport, are protected from obesity-associated inflammation and insulin resistance [18]. However, these experiments did not specifically address the role of insulin action, as CAP is a scaffold protein implicated both in insulin signaling and also cytoskeleton regulation [45], [46].

The present results not only reveal clearly that IR-dependent signaling is critical for macrophage recruitment to WAT upon obesity development, they also define at least two potential mechanisms responsible for this phenomenon: increased apoptosis of macrophages and reduced tissue invasion by these cells due to decreased expression of MMP-9.

It is well documented that insulin can protect macrophages from apoptosis induced by numerous stimuli, such as serum/glucose deprivation, lipopolysaccharides and UV-irradiation *in vitro* [17], [20], [47]. More recently, Senokuchi *et al.* showed that insulin protects macrophages from ER stress-mediated apoptosis by free cholesterol in the development of atherosclerosis [20], [48]. Consistent with the latter notion, we find that saturated fatty acids, such as palmitate, which are increased in the circulation of obese patients [49], can result in macrophage apoptosis and that this effect is inhibited by insulin *in vitro*. However, we could not observe increased macrophage apoptosis in adipose tissue or altered numbers of circulating monocytes in obese IR^Δmyel^-mice, questioning the *in vivo* relevance of this finding.

Aside from regulation of macrophage apoptosis, we find that insulin promotes expression and activation of MMP-9 in these cells, a protease involved in tissue invasion by macrophages [25]. Furthermore, we could directly demonstrate that siRNA-mediated ablation of MMP-9 drastically impairs macrophage transmigration through a gelatin matrix and that this can be phenocopied by loss of the insulin receptor. Consistent with these results, it has been demonstrated that insulin augments MMP-9 in human monocytes *in vitro* [50] and that degradation of extracellular matrix (ECM) components by MMP-9 represents a key step during macrophage tissue invasion [51]. Indeed, reduced MMP-9 activity diminishes macrophage trans-ECM migration and protects from local inflammation and inflammation-associated cardiovascular disease [25]. Interestingly, San José et al. have recently demonstrated that insulin activates MMP-9 in murine macrophages in a PI3K/PKC-dependent manner through stimulation of the NADPH oxidase system [52]. Here, the authors propose that insulin-dependent MMP-9 activation might contribute to plaque instability in atherosclerotic lesions. Taking that into account, our results underline the important role of insulin receptor-dependent regulation of MMP-9 in macrophages and further extend it to another hyperinsulinemia-related disease state i.e. the development of obesity-associated inflammation and insulin resistance.

Notably, the LysMCre transgene mediates recombination of loxP-flanked alleles not only in macrophages but also in other myeloid lineage-derived cell types [53]. Therefore, one key question remains why, in our model, the reduction of adipose tissue infiltration is specific for macrophages while marker expression of other immune cell types e.g. granulocytes, T-lymphocytes and mast cells was unchanged. This might be due to the time-dependent fashion in which different subsets of immune cells invade the adipose tissue over the course of obesity. While adipose tissue granulocytes and T-lymphocytes already appear after 7 days and 6 weeks, respectively [13], [24], macrophage numbers do not significantly increase before 12–16 weeks of high fat feeding [7], [13]. Therefore, we cannot exclude that, despite macrophages, adipose tissue numbers of distinct immune cell subsets may be changed at different stages of obesity in our model. Additionally, immune cells invading the adipose tissue, especially macrophages and T-lymphocytes, can be further divided into distinct subtypes which are characterized by differential expression of specific surface markers [13], [54], [55]. Thus, analysis of adipose tissue immune cell populations by fluorescence activated cell sorting (FACS) could potentially yield a higher resolution of adipose tissue inflammatory cell composition than the quantitative realtime PCR analysis performed in this study. Nevertheless, our experiments indicate that protection from diet-induced insulin resistance appears to be primarily paralleled by reduced WAT-macrophage recruitment.

Another question is why reduced macrophage accumulation in obese IR^Δmyel^-mice is restricted to adipose tissue, while no significant difference of F4/80 expression could be observed in liver and skeletal muscle of these animals. This might be explained by our finding that in wildtype mice the obesity-induced infiltration of macrophages into adipose tissue is several magnitudes higher (∼20-fold vs NCD) than into liver and skeletal muscle (max. 2-fold). Therefore, the effect of general macrophage-autonomous impairment of migration ability may be particularly predominant in adipose tissue compared to other insulin target tissues.

In conclusion, our study directly demonstrates that, despite its positive effects on glucose metabolism in target tissues such as liver, skeletal muscle and WAT, *in vivo* insulin can also play a deleterious role during the development of the metabolic syndrome by its actions in cells of the innate immune response system. The molecular mechanism of how the insulin receptor signaling pathway affects macrophage function remains to be further defined, but the present study suggests that this may offer a site for pharmacological intervention that could lead to novel therapeutic strategies for metabolic diseases.

## Methods

### Animals

All animal procedures were conducted in compliance with protocols approved by local government authorities and were in accordance with NIH guidelines. Mice were housed in groups of 3–5 at 22–24°C in a 12:12 h light/dark cycle with lights on at 6 a.m. Animals were either fed a normal chow diet (Teklad Global Rodent # T.2018.R12; Harlan, Germany) containing 53.5% of carbohydrates, 18.5% of protein, and 5.5% of fat (12% of calories from fat) or from week 4 of age a high fat diet (# C1057; Altromin, Germany) containing 32.7%, 20% and 35.5% of carbohydrates, protein and fat (55.2% of calories from fat), respectively. Water was available *ad libitum* and food was only withdrawn if required for an experiment. Body weight was measured once a week. Genomic DNA was isolated from tail tips, genotyping was performed by PCR. All experiments on mice were performed at 16 weeks of age.

### Generation of mice

*LysMCre* mice were mated with *IR^lox/lox^* mice, and a breeding colony was maintained by mating *IR^lox/lox^* with *LysMCre-IR^lox/lox^* mice. *IR^lox^* mice had been backcrossed for at least 5 generations on a C57BL/6 background, and *LysMCre* mice – initially established on a C57BL6/129sv background – had been backcrossed for 10 generations on a C57BL6 background before intercrossing them with *IR^lox^* mice. Only male animals from the same mixed background strain generation were compared to each other. *LysMCre* mice were genotyped by PCR as previously described [53]. *IR^lox/lox^* mice were genotyped by PCR with primers crossing the loxP site as previously described [26].

### Body composition

Body fat content was measured *in vivo* by nuclear magnetic resonance using a minispec mq7.5 (Bruker Optik, Ettlingen, Germany) as previously described [56].

### Indirect calorimetry and food intake

All measurements were performed in a PhenoMaster System (TSE systems, Bad Homburg, Germany), which allows measurement of metabolic performance. Mice were placed at room temperature (22°C–24°C) in 7.1-l chambers of the PhenoMaster open circuit calorimetry. Mice were allowed to adapt to the chambers for at least 24h. Food and water were provided *ad libitum* in the appropriate devices and measured by the build-in automated instruments. Parameters of indirect calorimetry and food intake were measured for at least the following 48 hr. Presented data are average values obtained in these recordings.

### Glucose and insulin tolerance tests

Glucose tolerance tests were performed after a 16–17h fasting period. After determination of fasted blood glucose levels, each animal received an i.p. injection of 20% glucose (10ml/kg) (DeltaSelect, Germany). Blood Glucose levels were detected after 15, 30, 60 and 120 minutes. Insulin tolerance tests were performed with mice fed *ad libitum*. After determination of basal blood glucose levels, each animal obtained an i.p. injection of insulin, 0.75U/kg (Actrapid; Novo Nordisk A/S, Denmark), and blood glucose was measured 15, 30 and 60 minutes after insulin injection.

### Euglycemic, hyperinsulinemic clamp studies

#### Catheter implantation

At the age of 16 weeks, male mice were anesthetized by intraperitoneal injection of avertin and adequacy of the anesthesia was ensured by the loss of pedal reflexes. A Micro-Renathane catheter (MRE 025; Braintree Scientific Inc., MA, USA) was inserted into the right internal jugular vein, advanced to the level of the superior vena cava, and secured in its position in the proximal part of the vein with 4-0 silk; the distal part of the vein was occluded with 4-0 silk. After irrigation with physiological saline solution, the catheter was filled with heparin solution and sealed at its distal end. The catheter was subcutaneously tunneled, thereby forming a subcutaneous loop, and exteriorized at the back of the neck. Cutaneous incisions were closed with a 3-0 silk suture and the free end of the catheter was attached to the suture in the neck as to permit the retrieval of the catheter on the day of the experiment. Mice were intraperitoneally injected with 1 ml of saline containing 15µg/g body weight of tramadol and placed on a heating pad in order to facilitate recovery.

#### Clamp experiment

Only mice that had regained at least 90% of their preoperative body weight after 6 days of recovery were included in the experimental groups. After starvation for 15 hours, awake animals were placed in restrainers for the duration of the clamp experiment. After a D-[3-^3^H]Glucose (Amersham Biosciences, UK) tracer solution bolus infusion (5µCi), the tracer was infused continuously (0.05µCi/min) for the duration of the experiment. At the end of the 40-minute basal period, a blood sample (50µl) was collected for determination of the basal parameters. To minimize blood loss, red blood cells were collected by centrifugation and reinfused after being resuspended in saline. Insulin (human regular insulin; NovoNordisc Pharmaceuticals, Inc., NJ, USA) solution containing 0.1% BSA (Sigma-Aldrich, Germany) was infused at a fixed rate (4µU/g/min) following a bolus infusion (40µU/g). Blood glucose levels were determined every 10 minutes (B-Glucose Analyzer; Hemocue AB, Sweden) and physiological blood glucose levels (between 120 and 150 mg/dl) were maintained by adjusting a 20% glucose infusion (DeltaSelect, Germany). Approximately 60 minutes before steady state was achieved, a bolus of 2-Deoxy-D-[1-^14^C]Glucose (10µCi, Amersham) was infused. Steady state was ascertained when glucose measurements were constant for at least 30 min at a fixed glucose infusion rate and was achieved within 100 to 130 min. During the clamp experiment, blood samples (5µl) were collected after the infusion of the 2-Deoxy-D-[1-^14^C]Glucose at the time points 0, 5, 15, 25, 35 min etc. until reaching the steady state. During the steady state, blood samples (50µl) for the measurement of steady state parameters were collected. At the end of the experiment, mice were killed by cervical dislocation, and brain, WAT and skeletal muscle tissues were dissected and stored at −20°C. Assays. Plasma [3-^3^H]Glucose radioactivity of basal and steady state samples was determined directly after deproteinization with 0.3M Ba(OH)_2_ and 0.3M ZnSO_4_ and also after removal of ^3^H_2_O by evaporation, using a liquid scintillation counter (Beckmann, Germany). Plasma Deoxy-[1-^14^C]Glucose radioactivity was directly measured in the liquid scintillation counter. Tissue lysates were processed through Ion exchange chromatography columns (Poly-Prep^R^ Prefilled Chromatography Columns, AG^R^1-X8 formate resin, 200–400 mesh dry; Bio Rad Laboratories, CA, USA) to separate 2-Deoxy-D-[1-^14^C]Glucose (2DG) from 2-Deoxy-D-[1-^14^C]Glucose-6-Phosphate (2DG6P).

#### Calculations

Glucose turnover rate (mg×kg^−1^×min^−1^) was calculated as the rate of tracer infusion (dpm/min) divided by the plasma glucose-specific activity (dpm/mg) corrected for body weight. HGP (mg×kg^−1^×min^−1^) was calculated as the difference between the rate of glucose appearance and glucose infusion rate. *In vivo* glucose uptake for each tissue (nmol×g^−1^×min^−1^) was calculated based on the accumulation of 2DG6P in the respective tissue and the disappearance rate of 2DG from plasma as described previously [57].

### Isolation of murine macrophages

#### Peritoneal macrophages

Mice were injected intreaperitoneally with 2 ml thioglycollate medium (4% in PBS). On day 4 *post* injection, the animals were sacrificed by CO_2_ anesthesia and cells were collected by peritoneal lavage with sterile PBS. After several washing steps, cells were resuspended in RPMI 1640 (supplemented with 10% FCS, 1% glutamine, 1% penicillin-streptomycin) and plated at a density of 10^6^ cells/ml.

#### Bone marrow–derived macrophages

Mice were sacrificed by CO_2_ anesthesia, rinsed in 70% (v/v) ethanol and bone marrow was isolated from femurs and tibias. After several washing steps, bone marrow cells were resuspended in IMDM (supplemented with 10% FCS, 1% glutamine, 1% penicillin-streptomycin and 10 ng/ml recombinant M-CSF (Peprotech)). Bone marrow cells were plated at a concentration of 1–2×10^6^ cells/ml in IMDM (supplemented with 10% FCS, 1% glutamine, 1% penicillin-streptomycin and 10 ng/ml recombinant M-CSF) on 10 cm bacterial petridishes and differentiated for 7–10 days. Preceding all the experiments, macrophages were washed two times with sterile PBS and, if stimulated with insulin, serum-starved for 16–20 h. Palmitate media was prepared as previously described [58].

### siRNA transfections

650 pmole siRNA (Silencer Select siRNA negative control, #4390846; Insr, #S68367; MMP9, #S69944; Applied Biosystems, CA, USA) were transferred to a 4-mm cuvette (Bridge, Providence, RI) and incubated for 3 minutes with 4×10^6^ bone marrow-derived macrophages (BMDM) in 100 µL Optimem (Invitrogen, Frederick, MD) before electroporation in a Gene Pulser X cell + CE module (Bio-Rad, Hercules, CA). Pulse conditions were square wave, 1000 V, 2 pulses, and 0.5-ms pulse length. 72–96 hours after electroporation, RNAi efficiency was tested using quantitative realtime PCR and silenced BMDM were used for functional assays.

### Analytical procedures

Blood glucose levels were determined from whole venous blood using an automatic glucose monitor (GlucoMen GlycÓ; A. Menarini Diagnostics, Italy). Leptin, insulin, TNF-α, adiponectin and MMP-9 levels in serum were measured by ELISA using mouse standards according to manufacturer's guidelines (Mouse Leptin ELISA; Crystal Chem, IL, USA / Mouse Insulin ELISA; Crystal Chem, IL, USA / Mouse Adiponectin (HMW & total) ELISA; Alpco, NH, USA / Mouse TNF-α/TNFSF1A and MMP-9 (total) Quantikine ELISA Kit; R&D Systems, Inc., MN, USA). Serum FFAs were determined by colorimetric assay according to manufacturer's guidelines (NEFA kit; Wako chemicals GmbH, Neuss, Germany).

### Western blot analysis

Protein isolation from cells and tissues was performed as previously described [26]. Western blot analysis was performed as previously described [26] with antibodies raised against insulin receptor β subunit (IRβ, catalog # sc-711; Santa Cruz Biotechnology Inc.) and Akt (catalog # 9272; Cell Signaling) as a loading control. SAPK/JNK Kinase assay (catalog # 9810; Cell Signaling Technology Inc.) was performed following the manufacturers instructions. Western blot analysis of total JNK input was performed with an antibody raised against JNK (catalog # 9252; Cell Signaling Technology Inc.). Quantification of changes in optical density was performed with Quantity One (Bio-Rad Laboratories, München, Germany).

### Gelatin zymography

Gelatin zymography was performed as previously described [59]. Briefly, cell culture supernatants and tissue extracts were purified from lower molecular weight proteins (<50 kDa) by centrifugation through Microcon YM-50 Centrifugal Filter Units (Millipore, Billerica, MA, USA). 10–40 µg of protein were separated on SDS polyacrylamide gels (containing 0.1 mg/ml gelatine). Gels were renatured in 2.5% Triton X-100 followed by incubation in MMP activation buffer (50 mM Tris-HCl, 5 mM CaCl_2_, pH 8) at 37°C overnight in a humidified chamber. Gels were stained with 2.5 g/l Coomassie brilliant-blue R-250. Destaining was carried out with 40% (v/v) methanol until the bands appeared clearly.

### Isolation of adipocytes and stromal vascular fraction

Animals were sacrificed and epididymal fat pads were removed under sterile conditions. Adipocytes were isolated by collagenase (1 mg/ml) digestion for 45 min at 37°C in DMEM/Ham's F-12 1∶1 (DMEM/F12) containing 1% BSA. Digested tissues were filtered through sterile 150-µm nylon mesh and centrifuged at 250×g for 5 min. The floating fraction consisting of pure isolated adipocytes was then removed and washed three more times before proceeding to experiments. The pellet, representing the stromal vascular fraction containing preadipocytes, macrophages and other cell types, was resuspended in erythrocyte lysis buffer consisting of 154 mM NH_4_Cl, 10 mM KHCO_3_, and 0.1 mM EDTA for 10 min.

### Analysis of gene expression

RNA was isolated from cells and tissues using the Qiagen RNeasy Kit (Qiagen, Germany). The RNA was reversely transcribed with EuroScript Reverse Transcriptase (Eurogentec, Belgium) and amplified using TaqMan Universal PCR-Master Mix, NO AmpErase UNG with TaqMan Assay-on-demand kits (Applied Biosystems, CA, USA). Relative expression of target mRNAs (Adiponectin Mm00456425_m1, Arg1 Mm00475988_m1, Bcl2 Mm00477631_m1, Bax Mm00432050_m1, Ccl2 Mm00441242_m1, Ccl3 Mm00441258, Ccl5 Mm01302428_m1, Cxcl5 Mm00436451_g1, CD11c Mm00498698_m1, CD3 Mm00442746_m1, CD34 Mm00519283_m1, CD4 Mm00442754_m1, CD8 Mm01182108_m1, F4/80 Mm00802530_m1, G6pase Mm839363_m1, Gr-1 Mm00459644_m1, Il1β Mm00434228_m1, Il6 Mm00446190_m1, Ifng Mm00801778_m1, Igf1r Mm00802841_m1, InsR Mm00439693_m1, Kit Mm00445212_m1, Leptin Mm00434759, Mmp2 Mm00439506_m1, Mmp9 Mm00442991_m1, Pck1 Mm00440636_m1, TNFα Mm00443258_m1) was determined using standard curves based on WAT and samples were adjusted for total mRNA content by hypoxanthine guanine phosphoribosyl transferase 1 (Hprt1 Mm00446968_m1) mRNA quantitative PCR. Calculations were performed by a comparative method (2^−ΔΔCT^). Quantitative PCR was performed on an ABI-PRISM 7900 HT Sequence Detector (Applied Biosystems, Germany). Assays were linear over 4 orders of magnitude.

### Apoptosis assay

For assessment of apoptosis in primary macrophages, the DeadEnd™ Fluorometric TUNEL system (Promega Corporation, Madison, WI, USA) was used. The protocol for adherent cells was carried out according to the manufacturer's instructions. Slides were mounted with Vectashield DAPI medium (Vector Laboratories Inc, Burlingame, CA, USA) and analyzed under a fluorescence microscope. Quantification of DAPI- and FITC-positive cells was performed using AxioVision 4.2 (Carl Zeiss MicroImaging GmbH, Oberkochen, Germany).

### Transwell migration assay

Chemotaxis of BMDM was quantified using transwell migration assays. Polycarbonate filters (Costar, Corning, 24-well, 8 µm pore size) were coated with gelatin (0.2%, Sigma) for 1h at room temperature or overnight at 4°C. BMDM (2×10^5^ cells in 300 µl IMDM/0,5% FCS) were placed in the upper compartment and subsequently incubated at 37°C/5% CO_2_ to adhere. After 1h, 100 ng/ml MCP-1 (Peprotech) was added to IMDM/0,5% FCS in the lower compartment. Control assays were performed without chemokine. After incubation for 4 h at 37°C/5% CO_2_, transmigrated cells were stained with DAPI and nuclei were counted under a fluorescence microscope.

### Immunohistochemistry

Immunohistochemistry was performed on paraffin sections as previously described [60]. Quantification of adipocyte size and Mac-2-positive area was performed with AxioVision 4.2 (Carl Zeiss MicroImaging GmbH, Oberkochen, Germany).

### Statistical methods

Data was analyzed for statistical significance using a two-tailed unpaired student's T-Test.

## Supporting Information

Figure S1 (A) Relative expression of F4/80 mRNA in untreated (basal) and palmitate (500 µM) stimulated bone marrow-derived macrophages of control- and IR^Δmyel^-mice. (n = 4 independent experiments; white bars represent control and black bars IR-deficient macrophages.) (B) Relative expression of F4/80 mRNA in liver and skeletal muscle (SM) of male C57Bl/6 mice fed either NCD or HFD for 22 weeks. (n = 8; white bars represent NCD and black bars HFD-fed animals.) (Results are means ± SEM; n.s. = not significant.)
(0.17 MB TIF)

Figure S2 Insulin receptor-deficient macrophages are prone to lipid-induced apoptosis. (A) TUNEL assay of bone marrow-derived macrophages of control- and IR^Δmyel^-mice (IR^−/−^) (representative pictures shown). Cells were left untreated (basal) or treated with insulin (50 ng/ml), palmitate (500 µM) or both for 24 h. (B) The percentage of TUNEL-positive cells (green) of the number of DAPI-positive nuclei (blue) was determined microscopically. (n = 4.) (C) Relative expression of Bax mRNA in bone marrow-derived macrophages of control- and IR^Δmyel^-mice. Cells were left untreated (basal) or treated with insulin (50 ng/ml), palmitate (500 µM) or both for 8 h. (n = 4.) (D) Relative expression of Bcl-2 mRNA in bone marrow-derived macrophages of control- and IR^Δmyel^-mice. Cells were left untreated (basal) or treated with insulin (50 ng/ml), palmitate (500 µM) or both for 8 h. (n = 4.) (Results are means ± SEM; white bars represent control and black bars represent IR-deficient macrophages *p≤0.05; **p≤0.01; ***p≤0.001; n.s. = not significant.)
(2.47 MB TIF)
