# Supplementary figures and images for: Myeloid Cell-Restricted Insulin Receptor Deficiency Protects Against Obesity-Induced Inflammation and Systemic Insulin Resistance
Source: PLoS Genet. 2010 May 6;6(5):e1000938. doi: 10.1371/journal.pgen.1000938 (PMC2865520; doi:10.1371/journal.pgen.1000938)

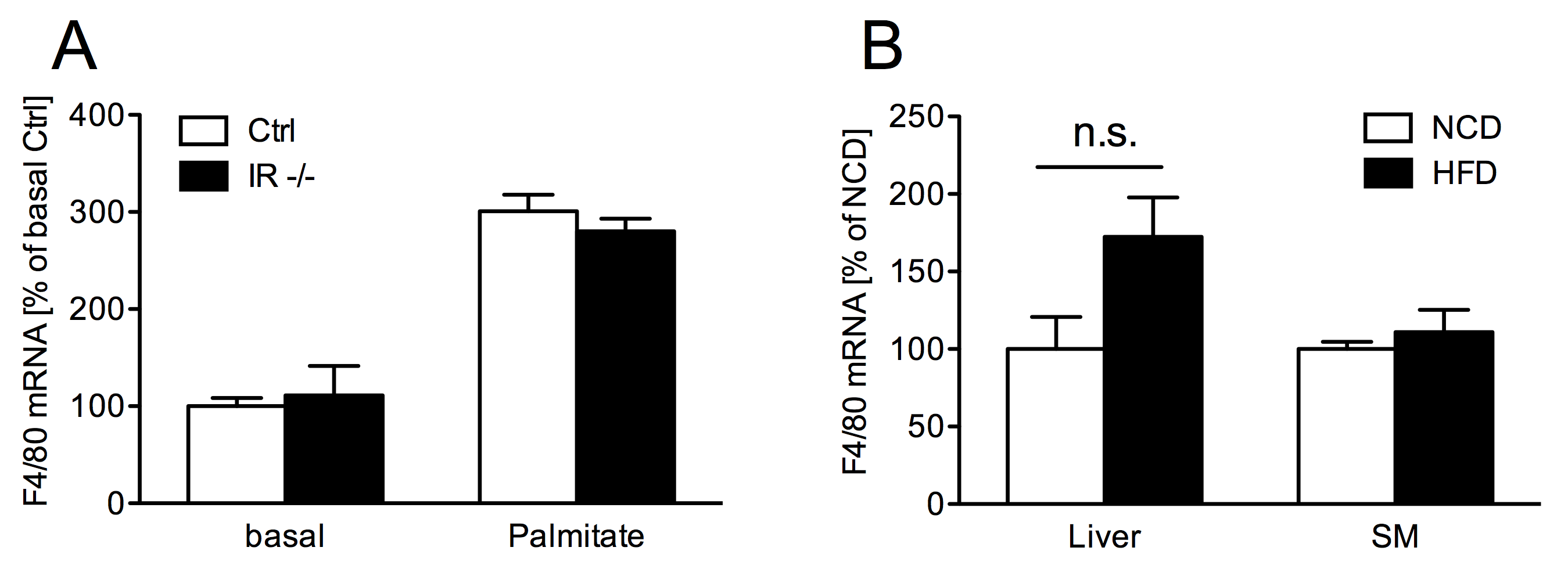

Supplement: Figure S1 — (A) Relative expression of F4/80 mRNA in untreated (basal) and palmitate (500 µM) stimulated bone marrow-derived macrophages of control- and IRΔmyel-mice. (n = 4 independent experiments; white bars represent control and black bars IR-deficient macrophages.) (B) Relative expression of F4/80 mRNA in liver and skeletal muscle (SM) of male C57Bl/6 mice fed either NCD or HFD for 22 weeks. (n = 8; white bars represent NCD and black bars HFD-fed animals.) (Results are means ± SEM; n.s. = not significant.) (0.17 MB TIF) [file pgen.1000938.s001.tif]

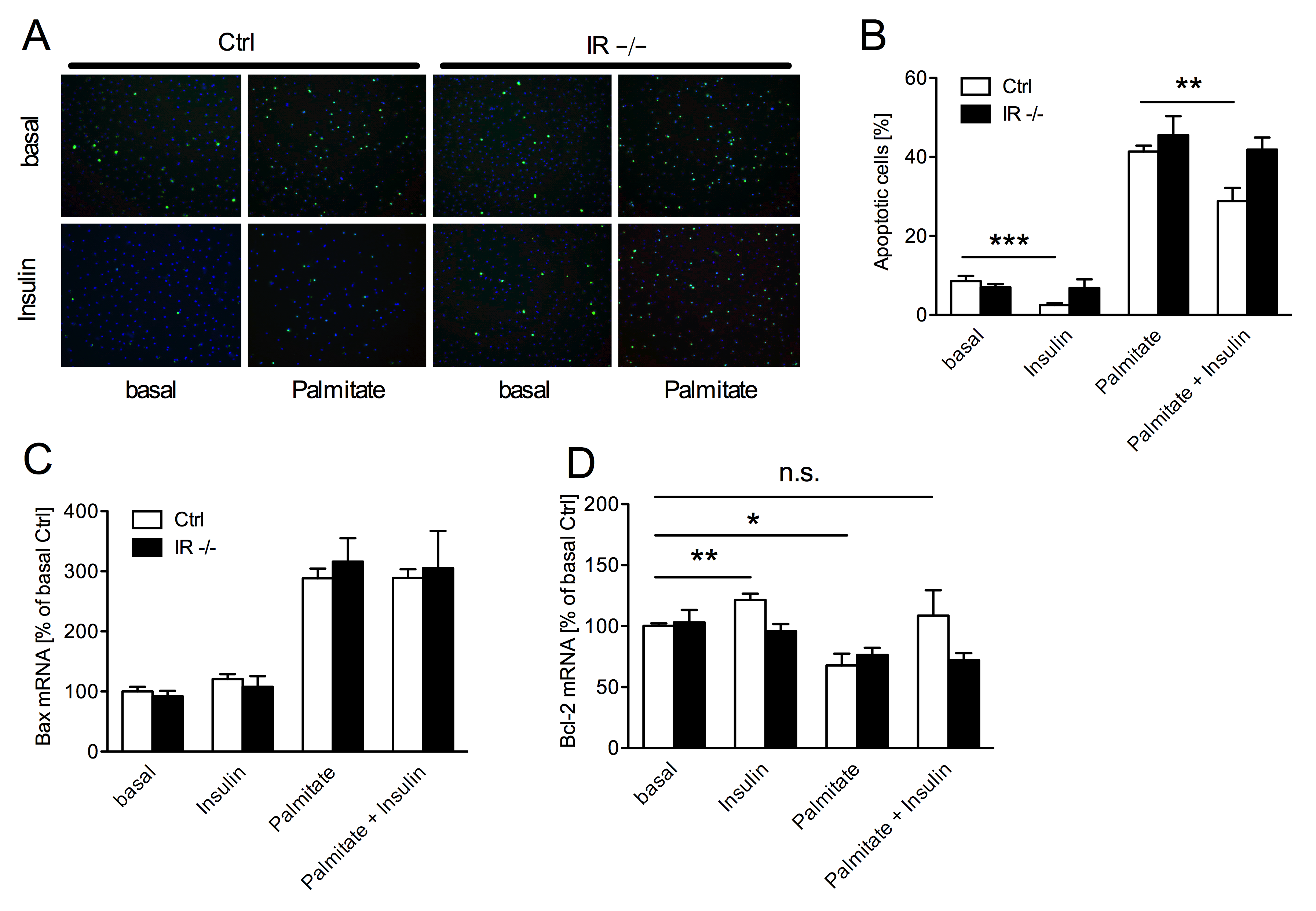

Supplement: Figure S2 — Insulin receptor-deficient macrophages are prone to lipid-induced apoptosis. (A) TUNEL assay of bone marrow-derived macrophages of control- and IRΔmyel-mice (IR−/−) (representative pictures shown). Cells were left untreated (basal) or treated with insulin (50 ng/ml), palmitate (500 µM) or both for 24 h. (B) The percentage of TUNEL-positive cells (green) of the number of DAPI-positive nuclei (blue) was determined microscopically. (n = 4.) (C) Relative expression of Bax mRNA in bone marrow-derived macrophages of control- and IRΔmyel-mice. Cells were left untreated (basal) or treated with insulin (50 ng/ml), palmitate (500 µM) or both for 8 h. (n = 4.) (D) Relative expression of Bcl-2 mRNA in bone marrow-derived macrophages of control- and IRΔmyel-mice. Cells were left untreated (basal) or treated with insulin (50 ng/ml), palmitate (500 µM) or both for 8 h. (n = 4.) (Results are means ± SEM; white bars represent control and black bars represent IR-deficient macrophages *p≤0.05; **p≤0.01; ***p≤0.001; n.s. = not significant.) (2.47 MB TIF) [file pgen.1000938.s002.tif]
